# Development and Characterization of an Antioxidant and Antimicrobial Film Composited by Hydroxyethyl Cellulose and Sulfated Rice Bran Polysaccharides for Food Packaging

**DOI:** 10.3390/foods13060819

**Published:** 2024-03-07

**Authors:** Bing-Jie Chen, Gui-Ge Liu, Xiao Wang, Hong-Ru Liu, Yi Zhang, Chun-Fang Wang, Chen-Xia Liu, Yong-Jin Qiao

**Affiliations:** 1 Institute of Crop Breeding and Cultivation, Shanghai Academy of Agricultural Science, Shanghai 201403, China; chenbingjie0204@126.com (B.-J.C.); wangxiao.0127@163.com (X.W.); hear2008dream@163.com (H.-R.L.); zhangyi_ppls@hotmail.com (Y.Z.); fhwcf@126.com (C.-F.W.); liuchenxia@saas.sh.cn (C.-X.L.); 2 College of Food Science and Technology, Shanghai Ocean University, Shanghai 201306, China; 13761411201@163.com

**Keywords:** hydroxyethyl cellulose, sulfated rice bran polysaccharides, film, antioxidant, bacteriostatic properties

## Abstract

The nonantimicrobial properties and relatively poor mechanical properties of hydroxyethyl cellulose (HEC) limit its use in packaging. Sulfated rice bran polysaccharides (SRBP) possess significant antioxidant and antimicrobial activities. The purpose of this study was to investigate the effect of different concentrations of SRBP on the physical and mechanical properties and the functional characteristics of HEC/SRBP films. The physical properties of the HEC/20% SRBP films, such as water resistance, water vapor barrier, light barrier, and tensile strength, improved significantly (*p* < 0.05) compared with those of the HEC films. Scanning electron microscopy and Fourier transform infrared spectrometry showed that HEC formed hydrogen bonds with SRBP and exhibited better compatibility. Thermogravimetric analysis revealed that the addition of SRBP was beneficial to the thermal stability of the films. In addition, the antioxidant and bacteriostatic properties of the films were enhanced by the addition of SRBP to HEC, with the 20% SRBP films showing the most significant enhancement in activity. Therefore, the HEC/20% SRBP films show potential for development for use as active food packaging.

## 1. Introduction

Petroleum-based polymer packaging materials are widely used owing to their low cost, easy production, and good barrier properties. The extensive use of these nonbiodegradable packaging materials not only leads to many health problems but is also responsible for an increase in serious problems due to environmental pollution. Utilizing renewable resources to develop green and environmentally friendly biodegradable packaging materials could help alleviate the environmental problems resulting from the use of petroleum-based plastics, and this area is now a research hotspot worldwide in the field of packaging [1]. Hydroxyethyl cellulose (HEC) is a cellulose derivative that has been widely applied in many fields owing to its good film-forming characteristics, biodegradability, biocompatibility, odorless nature, and nontoxicity [2]; however, the toughness, ductility, and loading properties of HEC are poor and need further enhancement by compounding it with other substances [3]. Huang et al. [4] added nanofibers to synthesize HEC membranes with improved ultraviolet (UV) resistance and mechanical properties. The addition of carboxymethyl chitosan and zinc oxide enhanced the solvent resistance, maximum load-bearing capacity and UV shielding ability of HEC films [3].

Polysaccharides are an important and promising source for the development of renewable green materials owing to their thermoplasticity, abundant availability, low price, and better functional properties [5]. Studies have confirmed that polysaccharides can make up for film defects, e.g., the addition of chickpea husk polysaccharides to carboxymethyl cellulose resulted in enhanced mechanical properties and thermal stability of the films [6]. The addition of coffee ground polysaccharides to carboxymethyl cellulose improved transparency and significantly increased the light barrier properties of the synthesized films [7].

Rice bran is the outer tissue of brown rice and is mainly composed of pericarp, aleurone layer, and germ, accounting for approximately 7% of the overall weight of rice. It is an important byproduct during the milling of brown rice. Over the past decade, the integrated utilization of rice bran has been very low, especially in developing countries, where they are mainly used as feed or discarded, leading to a great waste of valuable resources [5]. Much research has reported that rice bran is full of biologically active substances that have nutritional benefits such as antioxidant, lipid-lowering, and anti-inflammatory properties [8]. Rice bran polysaccharide (RBP), as an important active substance in rice bran, has antioxidant and antibacterial functions [5], and it has been found that sulfate modification can change their nature and structure and improve its biological activities [9]. Our preliminary study verified that the antimicrobial and antioxidant activities of RBP were enhanced by sulfation modification. Therefore, this study explores the potential of HEC/sulfated RBP (SRBP) films as an active packaging material by investigating the physical and chemical properties as well as the antioxidant and antimicrobial activities of the composite HEC/SRBP film.

## 2. Materials and Methods

### 2.1. Materials 

Dimethyl sulfoxide, sulfur trioxide pyridine complex, and HEC were supplied by the Shanghai Yuanye Co., Ltd. (Shanghai, China). LB broth culture medium and Na culture medium was supplied by the Shanghai Sangong Bioengineering Co., Ltd. (Shanghai, China). *Escherichia coli* (*E. coli*, CGMCC, 1.0907) and *Staphylococcus aureus* (*S. aureus*, CGMCC, 1.0089) were provided by the microbiology laboratory of Ningbo University (Ningbo, China).

### 2.2. Preparation of RBP

RBP was prepared from rice bran following the water extraction and alcohol precipitation method described by Liu et al. [10]. Briefly, defatted rice bran (100 g) was added to distilled water (2000 mL) and extracted at 90 °C for 2 h. The extract was filtered and the above operation was repeated. α-Amylase and papain were added to the mixture to remove starch and proteins. The mixed solution was precipitated with 95% ethanol. The precipitate was dissolved and dialyzed for 3 days. Subsequently, it was lyophilized to produce RBP.

### 2.3. Preparation of SRBP

SRBP was prepared by referring to the procedures published by Liu et al. [10] and Li et al. [11] with slight revisions. RBP (600 mg) was mixed with 90 mL of dimethyl sulfoxide and stirred for 1 h at 25 °C to completely dissolve the RBP. Next, sulfur trioxide pyridine complex (20 times RBP, *w*/*w*) was added and allowed to react at 55 °C for 2 h. After cooling, 1 M NaOH was used to neutralize the reaction solution and then dialyzed. Lastly, the solution was concentrated and lyophilized to obtain SRBP.

### 2.4. Preparation of the Composite Film

HEC (1%) was stirred at 80 °C for 1 h and cooled, followed by the addition of glycerol (plasticizer, 30% of HEC, *w*/*w*) and various concentrations of SRBP (5%, 10%, 15%, and 20% of HEC). The solutions were stirred at 25 °C for 0.5 h, degassed in a vacuum for 1 h, poured into a container (20 mL), and dried at 60 °C for 16 h. Finally, the HEC/SRBP films could be peeled off and kept in a closed reservoir at 50% relative humidity (RH) and a constant temperature (23 ± 2 °C) for 3 days. The films with different concentrations of SRBP were named 5% SRBP, 10% SRBP, 15% SRBP, and 20% SRBP; the film without SRBP was named HEC and served as the control film.

### 2.5. Physical Characteristics of Films

#### 2.5.1. Thickness

Referring to the method of Chen et al. [12], the film thickness was obtained by measuring 10 random points of the film using a micrometer (Mitutoyo, Digimetic caliper, Kanagawa, Japan) and calculating the average.

#### 2.5.2. Moisture Content and Water Solubility 

Film samples (W_1_) were dried at 105 °C to a constant weight (W_2_) and the moisture sample was calculated using Equation (1). We completely immersed the sample (W_2_) in 100 mL of distilled water and stirred for 24 h. The weight of the film (W_3_) was obtained after drying the film to a constant weight, and the water solubility was calculated using Equation (2).

(1) $$Moisture\;content\;\left(\%\right)=\frac{\left(W_{1}-W_{2}\right)}{W_{1}}\times 100$$

where W_1_ and W_2_ represent the weight of the film before and after drying, respectively.

(2) $$Water\;solubility\;\left(\%\right)=\frac{\left(W_{2}-W_{3}\right)}{W_{3}}\times 100$$

where W_2_ and W_3_ are the dry weights of the film before and after immersion, respectively.

#### 2.5.3. Water Vapor Permeability (WVP)

The WVP of the films was evaluated using the protocol published by Akhta et al. [6].

#### 2.5.4. Color Property and Transparency

We determined the color characteristics of the films using a colorimeter (Spectrophotometer CM-5, Konica Minolta Holdings Ltd., Tokyo, Japan) following the method published by Li et al. [13]. A white standard plate was used for calibration (L* = 93.49 [lightness], a* = 0.25 [redness/greenness], and b* = 0.09 [yellowness/blueness]). *L*, *a*, and *b* were measured at six randomly chosen points of the film and the total color difference (Δ*E*) was calculated using Equation (3). Transparency: Films were synthesized and their absorbance was measured at 600 nm using ultraviolet visible spectroscopy (UV2450, Shimadzu Co., Ltd., Tokyo, Japan) and calculated using Equation (4).

(3) $$\Delta E=\sqrt{{\left(L^{*}-L\right)}^{2}+{\left(a^{*}-a\right)}^{2}+{\left(b^{*}-b\right)}^{2}}$$

where L*, a*, b* are the standard values and *L*, *a*, *b* are the sample values.

(4) $$Transparency\;value=-\frac{\log\left(T600\right)}{t}$$

where *T*600 is the transparency at 600 nm and *t* is the film thickness (mm).

#### 2.5.5. Mechanical Properties: Tensile Strength (TS) and Elongation at Break (EB)

The TS and EB were determined using a texture analyzer (TA.XT Plus, Stable Micro Systems Ltd., Godalming, UK) following the method adopted by Rui et al. [14].

#### 2.5.6. Morphological Characteristics

The morphological properties of the samples were observed using scanning electron microscopy (SEM; TM 4000 plus, Hitachi Ltd., Tokyo, Japan) at a magnification of 100× and 500×.

#### 2.5.7. Fourier Transform Infrared Spectrometry (FT-IR)

The FT-IR spectra of the films were recorded in the infrared range of 4000–500 cm^−1^ using an FT-IR spectrometer (Nicolet iS20 FT-IR, Thermo Fisher Scientific Inc., Waltham, MA, USA). The spectra were collected in 32 scans at a resolution of 4 cm^−1^ for each sample.

### 2.6. Thermodynamic Analysis

The film samples (15 mg) were heated from 20 °C to 200 °C at a rate of 5 °C/min in a nitrogen atmosphere using a differential scanning calorimeter (DSC, Q2000, TA Instruments, New Castle, DE, USA) [15].

Referring to the method of Zhou et al. [16], the film samples (5–10 mg) were placed in a crucible for thermogravimetric analysis (TGA 500, TA Instruments Inc., New Castle, DE, USA). The reaction conditions were as follows: after equilibration at 30 °C for 5 min, the samples were heated to 800 °C at a rate of 10 °C/min in a nitrogen atmosphere, and the thermal weight loss thermograms of the samples were recorded.

### 2.7. Antioxidant Activities of the Films

#### 2.7.1. 1,1-Diphenyl-2-picrylhydrazyl (DPPH) Radical Scavenging Activity

DPPH radical scavenging activity was evaluated according to the procedure described by Doh et al. [17].

#### 2.7.2. Superoxide Anion Radical (O_2_^−^) Scavenging Activity

The protocol published by Li et al. served as the method for assessing the rate of O_2_^−^ scavenging [18]. 

### 2.8. Antibacterial Activity

We took out the preserved strain (*E. coli*, CGMCC, 1.0907 and *S. aureus*, CGMCC, 1.0089) from a −80 °C refrigerator and thawed it, then dipped a small amount of bacterial liquid into the inoculating ring and marked the line on the NA medium, and incubated it at 37 °C for 16–18 h. We picked out a single colony and inoculated it into the LB broth culture medium, and then incubated it at 37 °C on a shaker for 18–24 h at 180 r/min, and then sucked up 0.5 mL of it and inoculated it into the LB broth culture medium again. The above diluted bacterial suspension (10^5^ CFU/mL) was added to NA that cooled to 50 °C, and the mixture was shaken slightly and then poured into the plate and left to solidify. Then, samples (8-mm discs) were incubated in a medium containing bacteria at 37 ± 1 °C for 24 h [19]. Polyethylene film served as a blank control. The diameter of inhibition (mm) around the film was measured as the inhibition circle.

### 2.9. Statistical Analysis

Origin 2018 software was applied for mapping and SPSS 22.0 was applied for processing experimental data. Data are presented as mean ± standard deviation. Duncan’s multiple comparison test was used to analyze the significance.

## 3. Results

### 3.1. Physical and Mechanical Properties of Films

From Table 1, it can be concluded that the film thickness increases with an increase in SRBP concentration, which may be due to the addition of SRBP to change the solid content, or the enhanced interaction between SRBP and HEC makes the two bind more tightly. Akhtar et al. have reported a comparable trend in their findings [6]. The film thickness was determined to be in the range of 0.0305–0.0522 mm, which is in accordance with the standards of the American Society for Testing and Materials [20].

Films containing SRBP showed lower moisture content compared with HEC films. With an increase in SRBP content (5%, 10%, 15%, and 20%), the moisture content of the films declined from 11.85% to 9.03%, 8.31%, 4.11%, and 3.51%, respectively (Table 1). This is because intermolecular hydrogen bonds are formed between the hydrophilic hydroxyl groups of HEC and the hydroxyl groups of SRBP, which may limit the hydrophilic interactions of HEC with water molecules, thereby leading to a reduction in water content [21].

It is well known that water-soluble materials have significant advantages in terms of preparation and recycling. Both HEC and SRBP are soluble in water; however, in the food-packaging industry, films with high water solubility are unsuitable for use with foods having a high water content [3]. The water solubility of the films reduced from 86.60 ± 0.66% (HEC group) to 64.38 ± 0.56% (20% SRBP group), indicating that the incorporation of SRBP improved the water resistance of the films (Table 1). This finding could be attributed to the intermolecular interactions between HEC and SRBP, wherein the molecular chains intertwine to form a complex, interpenetrating network structure. Moreover, the spatial site resistance between the hydrophilic groups improves the cohesion of the HEC matrix, thereby reducing water solubility [22]. Our findings were in accordance with those of Ballesteros et al. [7], who reported that the water content and water solubility of carboxymethyl cellulose/waste coffee grounds polysaccharide composite film decreased with increasing polysaccharide concentration.

Water vapor permeability (WVP) plays a vital role in food packaging. Films with a low WVP have good water barrier properties, which on the one hand reduces water loss from the food, and on the other hand inhibits the intrusion of external water vapor and reduces the risk of microbial growth, thus helping to extend the storage period of the food [23]. The WVP of the HEC film was 4.21 × 10^−10^ g/m s Pa, which decreased from 3.6810^−10^ g/m s Pa (5% SRBP) to 1.15 × 10^−10^ g/m s Pa (20% SRBP) with an increase in SRBP concentration, indicating that the addition of SRBP improved the WVP of the films (Table 1) [22]. This is mainly because the addition of SRBP made the diffusion path of gas molecules in the composite membrane more tortuous and complex, thereby decreasing the WVP and improving the barrier performance of the film. Additionally, an increase in membrane thickness led to an increase in the pressure required for the penetration of water vapor molecules [24].

Transparency, as a key factor affecting packaging performance, reflects the ability of the film to block UV light [25]. Table 1 shows that the light transparency decreases with increasing SRBP concentrations, indicating that the amount of light transmitted through the film is reduced. It is likely that the pigments in the polysaccharides absorb some of the light waves [26]. Low light transparency helps prevent oxidative deterioration, delaying the occurrence of undesirable phenomena such as food discoloration, rotting, and loss of nutrients [27]. Therefore, preservation films with added SRBP are more conducive to maintaining food quality.

### 3.2. Color Characteristics 

Color directly affects consumer acceptance and is an important parameter in the film-production process. Table 2 shows that HEC films had the highest brightness (*L*) value (78.34), indicating that HEC appears brighter compared with the films containing SRBP. The gradual increase in *a* and *b* values with an increasing SRBP concentration confirms that the color changes significantly from light transparent (HEC film) to light brown (HEC/RBP film). The Δ*E* of the film increases consistently from 18.39 ± 0.21 (HEC) to 69.01 ± 0.52 (20% SRBP), suggesting that the color of the composite film increases and deepens with increasing SRBP concentration. Ballesteros et al. have reported similar results [7].

### 3.3. Mechanical Properties

**Figure 1:**
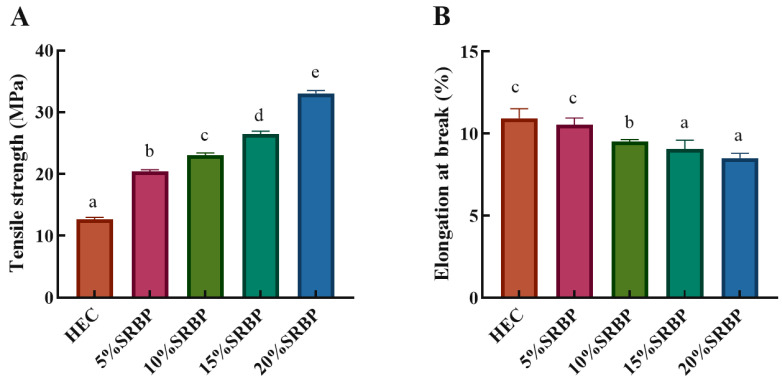
TS (**A**) and EB (**B**) of the HEC/SRBP films; results with different letters are significantly different (*p* < 0.05).

TS and EB are important parameters that characterize the mechanical properties of films [6], and the enhancement of the mechanical properties of films helps in improving their resistance to stress during transportation and storage, inhibiting the fracture of films and maintaining their integrity [6]. The TS of HEC/SRBP films increased from 12.66 ± 0.33 MPa to 33.04 ± 0.47 MPa when the SRBP content was increased from 0% to 20% (Figure 1A), indicating a significant increase compared with the film without SRBP. This finding confirmed that the incorporation of polysaccharides helps improve the TS, which, in turn, is attributed to the fact that HEC is a rigid molecular chain that can absorb stress; the addition of polysaccharides contributes to effective stress transfer. On the other hand, the hydroxyl and carboxyl groups on the polysaccharide chain and the hydroxyl group on the HEC chain form hydrogen bonds. When the polysaccharide content was higher, the compatibility between HEC and polysaccharides was superior, the number of hydrogen bonds increased, and the tensile strength of the composite film improved [1]. The EB decreased from 10.94 ± 0.58% to 8.57 ± 0.27% (Figure 1B), which was attributable to an increase in physical cross-linking interactions with increasing RBP, thus limiting the mobility of the HEC chains, and during further stretching, the stresses are concentrated, leading to the fracture of the HEC membrane [4].

### 3.4. SEM

**Figure 2:**
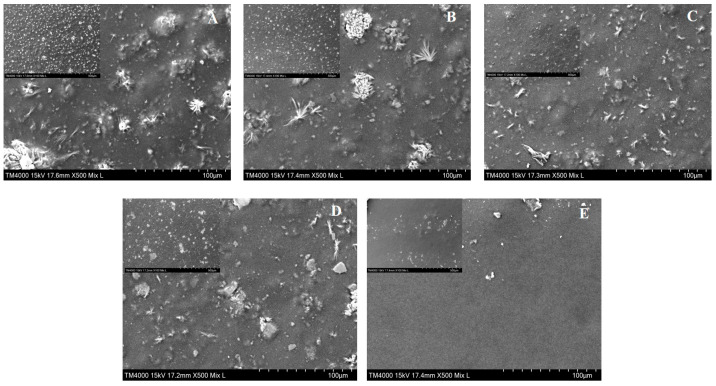
Morphological structure of HEC-based films blended with different SRBP. Note: (**A**–**E**) represent HEC films prepared with 0%, 5%,10%, 15% and 20% SRBP, respectively. Magnification: ×100 for small picture; ×500 for big picture.

SEM was used to characterize the smoothness, porosity, lamellar structure, and homogeneity of the film surface [28]. Both the control and composite film micrographs show smooth, dense structures without any voids or cracks (Figure 2). A previous study established that HEC films have a smooth, dense, and flat microstructure [3]. However, an equally flat and dense microstructure with white dots was observed in the HEC films in this study, which may be due to the migration of glycerol to the film surface during the drying process, resulting in an excessive accumulation of glycerol at the interface [16]. The dotted structure reduced gradually with an increase in SRBP, and was most obvious with 20% SRBP, indicating that the polysaccharide had high compatibility and good adhesion with HEC and glycerol [29].

### 3.5. FT-IR 

**Figure 3:**
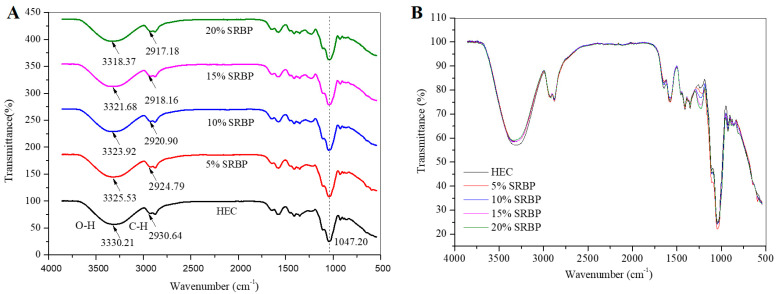
FT-IR spectra (**A**) and (**B**) of the HEC/SRBP films.

FT-IR is a rapid and nondestructive technique to study structural changes in composite films [4]. It was used to evaluate the intermolecular interactions between HEC and SRBP in HEC/SRBP films. As shown in Figure 3A,B, the same transmission bands were observed in all films, indicating that the HEC/SRBP films had the same structure and chemical bonding as the HEC films and that no new covalent bonds were formed between the hybrid films [22]. The peak at 2930.64 cm^−1^ corresponds to the saturated C-H stretching vibration, and a sharp peak can be observed at 1047.20 cm^−1^ in all films, indicating the presence of glycosidic bonds. The wider vibrational band between 3200 cm^−1^ and 3500 cm^−1^ could be attributed to the stretching vibration of the O-H group. The addition of SRBP flattened the peaks (Figure 3B), confirming that SRBP formed hydrogen bonds with HEC, thereby attenuating the stretching vibration of free O-H [30]. This finding was similar to the results reported by Lin et al. [31]. Compared with those in the HEC film, the absorption peaks of the O-H and C-H of the 20% SRBP film shifted from 3330.21 cm^−1^ and 2930.64 cm^−1^ to 3318.37 cm^−1^ and 2917.18 cm^−1^, respectively. Both of them were red-shifted to lower wavelengths, which may be attributed to hydrogen bond formation between SRBP and HEC and the enhancement of the intermolecular interactions [12]. Similar findings have been reported by Yuan et al. [26].

### 3.6. Thermodynamic Analysis 

**Figure 4:**
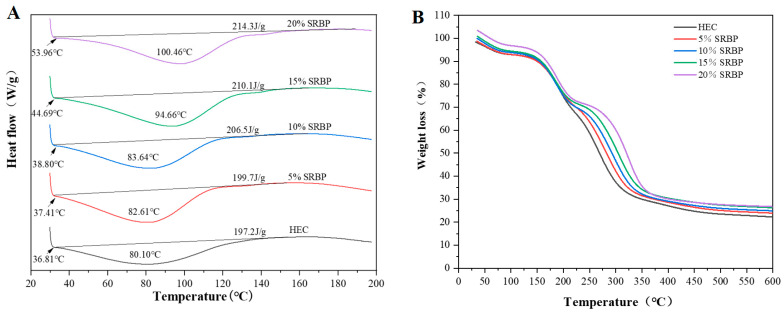
(**A**) Thermograms from differential scanning calorimetry (DSC); (**B**) Spectra from thermogravimetric analysis (TGA).

The peak area in the DSC curve indicates the amount of heat absorbed or released per unit mass of the sample to undergo phase transition. Table 3 shows the onset temperature (To), peak temperature (Td) and enthalpy change (ΔH) of the films. The higher the To, Td and ΔH are, the higher is the stability of the molecule [32]. As shown in Figure 4A, the curve shifted to a certain extent toward heat absorption at the beginning of the rise in temperature, probably due to the evaporation of water from the samples during testing. Both the onset and peak temperatures of the HEC/SRBP composite films shifted to higher temperatures with the increase in SRBP concentration. ΔH showed a similar trend: ΔH of the HEC film, 5% SRBP film, 10% SRBP film, 15% SRBP film, and 20% SRBP film was 197.2 J/g, 197.7 J/g, 206.5 J/g, 210.1 J/g, and 214.3 J/g, respectively, indicating a gradual increase in the thermal stability of the films. This is because HEC molecules contain three hydroxyl groups; therefore, the formation of intermolecular or intramolecular hydrogen bonds and crystalline structure is feasible. Chen et al. [12] prepared thymol/sodium alginate films and showed that the stability of the films was positively correlated with thymol concentration.

TGA is commonly used to characterize important information such as the thermal properties, thermal degradation, and weight loss of materials [1]. Next, the stability of the HEC/SRBP films was evaluated and the interaction between HEC and the polysaccharide was determined. Both HEC and SRBP are hydrophilic polymers containing -OH groups; therefore, they exhibit similar thermal degradation. Figure 4B shows four stages of weight loss. In the first stage (30–100 °C), the weight loss is small and relevant to the evaporation of water from the film; in the second stage (100–200 °C), the extent of weight loss increases rapidly, which is attributable to the loss of bound water and glycerol; in the third stage (200–350 °C), the maximum rate of mass loss of 40–45% is observed for all films, which is due to the degradation of the macromolecules HEC and polysaccharides; in the fourth stage (350–600 °C), the weight change plateaus as the material begins to carbonize. As the concentration of SRBP was increased, the temperature required for weight loss to occur in each stage of the film also increased, indicating that the addition of SRBP improved the thermal stability of the composite. This finding may be due to the increased interaction and crosslinking between SRBP and HEC, while at the same time having higher compatibility and forming a more stable structure. Similar findings have been published by Shivangi et al. [29].

### 3.7. Activity 

#### 3.7.1. Antioxidant Activity

**Figure 5:**
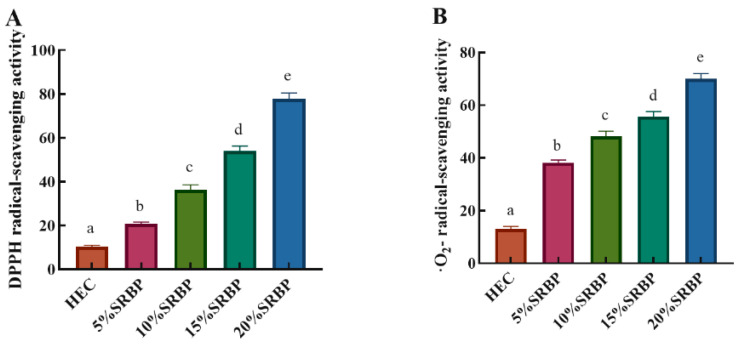
DPPH radical-scavenging activity (**A**) and·O_2_^−^ radical-scavenging activity (**B**) of the HEC/SRBP films, results with different letters indicate significant difference (*p* < 0.05).

Oxidation reactions not only cause food deterioration and loss of nutritional value but may also produce harmful substances; thus, the addition of natural antioxidants to food packaging materials has become a new means to extend the storage period of food [33]. DPPH is a stable free radical that has been widely used to assess antioxidant activity. Although a relatively weak oxidant, O_2_**^−^** can react with other molecules to generate stronger oxidizing substances, leading to oxidative damage and various diseases [34]. Therefore, the antioxidant properties of films can be assessed using DPPH and O_2_^−^ scavenging assays. The DPPH and O_2_^−^ radical scavenging rates of the HEC/SRBP films showed an increasing trend with an increase in SRBP content (Figure 5). The DPPH radical scavenging rate of the HEC/20% SRBP film increased from 10.34 ± 0.82% to 77.83 ± 1.12% and its O_2_^−^ radical scavenging rate increased from 13.00 ± 0.93% to 70.06 ± 1.34% compared with the control group, indicating that the incorporation of SRBP greatly increased the free-radical scavenging rate. Therefore, HEC/SRBP films with antioxidant properties can delay the oxidative deterioration of food, improve stability, and thus prolong the overall storage time. Studies have shown that several reducing hydroxyl groups in polysaccharides can react with free radicals, neutralize their activity, and exert an antioxidant effect [14]. Furthermore, the antioxidant properties of polysaccharides can be enhanced by modification via sulfation [35].

#### 3.7.2. Antibacterial Activity

**Figure 6:**
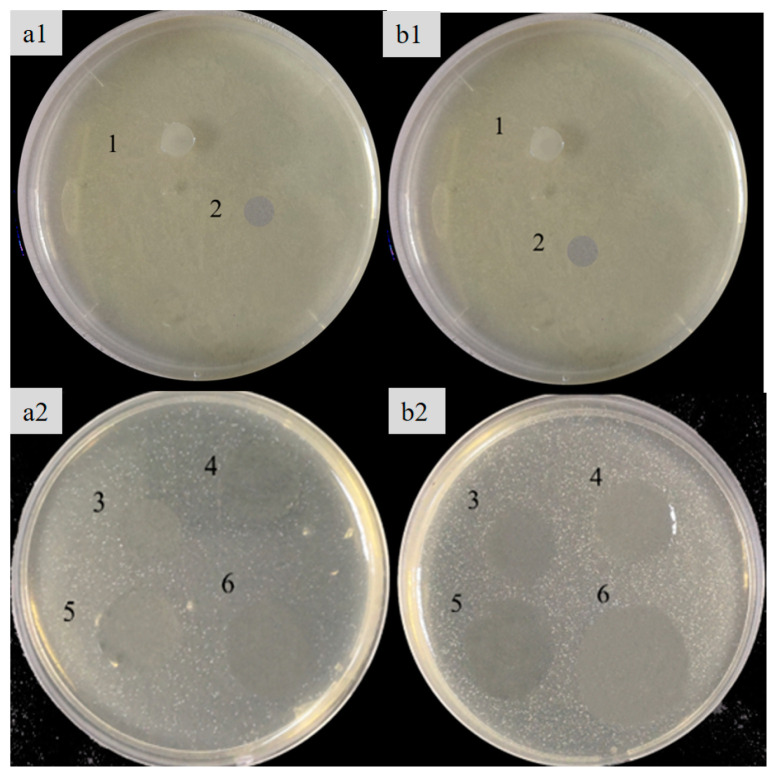
Inhibitory effects of the HEC/SRBP films on *Escherichia coli* (**a1**,**a2**) and *Staphylococcus au reus* (**b1**,**b2**). Note: 1: Polyethylene film; 2: HEC; 3: 5% SRBP; 4: 10% SRBP; 5: 15% SRBP; 6: 20% SRBP.

The bacteriostatic properties of the films were evaluated using the common susceptible bacteria in food, namely *E. coli* and *S. aureus*. The larger the circle of inhibition was, the stronger was the inhibitory effect on the bacteria. HEC had no inhibitory effect on both *S. aureus* and *E. coli* (0.00 ± 0.05 mm), whereas the composite film could inhibit the growth of both bacteria (Table 4 and Figure 6). The inhibitory effect was gradually enhanced with an increase in SRBP concentration, and an inhibition zone diameter around 8 mm diameter films for *E. coli* and *S. aureus* increased to 9.20 ± 0.10 mm and 10.23 ± 0.15 mm, respectively, when 20% of SRBP was used. We have demonstrated that SRBP has a better inhibitory effect on both *E. coli* and *S. aureus*, suggesting that the antimicrobial activity of the HEC/SRBP biofilm is attributed to the polysaccharide compounds. The antibacterial activity of the HEC/SRBP films against Gram-positive bacteria was better than that of Gram-negative bacteria, likely because of the outer lipid membrane in Gram-negative bacteria that serves as an additional barrier of protection and makes it more difficult to penetrate. On the other hand, Gram-positive bacteria lack an outer membrane. The cell wall of the cytoplasmic membrane, which consists of peptidoglycans, polysaccharides, phosphates, and proteins is more susceptible to the absorption of foreign substances [36]. These findings show that HEC/SRBP films have a bacteriostatic effect and can be effectively used as an antimicrobial packaging material in the food-preservation industry.

## 4. Conclusions

In this study, HEC-based composite biofilms with different concentrations of SRBP were successfully prepared. The moisture content, water solubility, water vapor permeability, and light transmittance of the films decreased gradually with an increase in SRBP concentration, indicating improvement in the water and UV barriers of the films. An increase in SRBP concentration led to a gradual increase in TS and a decrease in EB of the composite films. SEM revealed that the films had a smooth surface and dense internal structure. The red-shift of the O-H stretching absorption peaks in FT-IR spectroscopy was observed for HEC/SRBP, suggesting hydrogen bond formation between HEC and SRBP. Thermal analysis indicated a good interaction between HEC and SRBP, indicating that the thermal stability of the composite film has been improved. Moreover, the addition of SRBP enhanced the DPPH and O_2_^−^ radical-scavenging ability of the HEC/SRBP films as well as the inhibition of *E. coli* and *S. aureus*. Therefore, HEC/20% SRBP could be considered as a novel, environmentally friendly antimicrobial biofilm that can effectively protect packaged material from light, reduce water evaporation, prevent the oxidation and microbial contamination of food, and successfully extend the shelf life of food. In fact, this composite film has been applied to cherry tomatoes, confirming that the HEC/20% SRBP film can effectively retard the weight loss and deterioration of cherry tomatoes during the post-harvest refrigeration process and maintain their nutritional property and flavor profile [10].

But HEC/SRBP films have some limitations. Bioactive SRBP is hydrophilic and tends to swell in water, leading to rapid leakage of the active substance, which affects the freshness of the food, limiting its application in fresh meat, fresh-cut fruits, and other foods with high surface moisture content. Meanwhile, HEC/20% SRBP has a low elongation at the break and is easily damaged by external forces. The next step is to add, adjust and study the auxiliaries to make the membrane have various functions such as antimicrobial, antioxidant, and water resistance. Further, the membrane can be transformed from the monolayer membrane to the composite membrane by using the properties possessed by each component, highlighting the advantages of each component in forming the membrane, and minimizing or avoiding the disadvantages, so as to improve the performance of the membrane.

## Figures and Tables

**Table 1:** Physical and mechanical properties of films.

Film	Thickness (mm)	Moisture Content (%)	Water Solubility (%)	WVP × 10^−10^ (g/m s Pa)	Transparency (%)
Control (HEC)	0.0305 ± 0.002 ^a^	11.85 ± 0.21 ^e^	86.60 ± 0.66 ^e^	4.21 ± 0.11 ^e^	25.59 ± 0.34 ^e^
5% SRBP	0.0367 ± 0.001 ^b^	9.03 ± 0.13 ^d^	80.85 ± 0.73 ^d^	3.68 ± 0.23 ^d^	17.93 ± 0.26 ^d^
10% SRBP	0.0441 ± 0.002 ^b^	8.31 ± 0.15 ^c^	75.44 ± 0.49 ^c^	2.76 ± 0.16 ^c^	14.91 ± 0.11 ^c^
15% SRBP	0.0495 ± 0.003 ^c^	4.11 ± 0.35 ^b^	71.43 ± 0.62 ^b^	1.92 ± 0.28 ^b^	13.23 ± 0.15 ^b^
20% SRBP	0.0522 ± 0.001 ^c^	3.51 ± 0.28 ^a^	64.38 ± 0.56 ^a^	1.15 ± 0.19 ^a^	11.92 ± 0.21 ^a^

Values are means ± standard deviation (*n* = 3). Different letters in the same column indicate significant differences (*p* < 0.05).

**Table 2:** Color characteristics of films.

Film	*L*	*a*	*b*	Δ*E*
Control (HEC)	78.34 ± 0.31 ^e^	0.27 ± 0.09 ^a^	2.21 ± 0.11 ^a^	18.39 ± 0.21 ^a^
5% SRBP	63.21 ± 0.42 ^d^	3.38 ± 0.12 ^b^	9.56 ± 0.25 ^b^	34.82 ± 0.33 ^b^
10% SRBP	51.82 ± 0.25 ^c^	7.53 ± 0.28 ^c^	15.34 ± 0.37 ^c^	47.83 ± 0.35 ^c^
15% SRBP	46.38 ± 0.16 ^b^	9.43 ± 0.22 ^d^	19.38 ± 0.29 ^d^	54.54 ± 0.41 ^d^
20% SRBP	33.42 ± 0.37 ^a^	11.21 ± 0.32 ^e^	25.73 ± 0.46 ^e^	69.01 ± 0.52 ^e^

Values are means ± standard deviation (*n* = 3). Different letters in the same column indicate; significant differences (*p* < 0.05).

**Table 3:** DSC thermal parameters of films.

Films	T0 (°C)	Td (°C)	ΔH (J/g)
Control (HEC)	36.81 ± 0.11 ^a^	80.10 ± 0.14 ^a^	197.2 ± 0.16 ^a^
5% SRBP	37.41 ± 0.13 ^b^	82.61 ± 0.10 ^b^	199.7 ± 0.14 ^b^
10% SRBP	38.80 ± 0.12 ^c^	83.64 ± 0.15 ^c^	206.5 ± 0.12 ^c^
15% SRBP	44.69 ± 0.11 ^d^	94.66 ± 0.12 ^d^	210.1 ± 0.11 ^d^
20% SRBP	53.96 ± 0.12 ^e^	100.46 ± 0.11 ^e^	214.3 ± 0.13 ^e^

Values are presented as mean ± standard deviation (*n* = 3). Different lowercase letters (a–e) in the same column represent significant differences (*p* < 0.05).

**Table 4:** Inhibition zone diameter around 8 mm diameter films.

Film	*E. coli* (mm)	*S. aureus* (mm)
Polyethylene film	0.00 ± 0.05 ^a^	0.00 ± 0.05 ^a^
Control (HEC)	0.00 ± 0.05 ^a^	0.00 ± 0.05 ^a^
5% SRBP	3.11 ± 0.15 ^b^	5.40 ± 0.11 ^b^
10% SRBP	6.20 ± 0.10 ^c^	7.50 ± 0.10 ^c^
15% SRBP	7.13 ± 0.21 ^d^	9.35 ± 0.05 ^d^
20% SRBP	9.20 ± 0.10 ^e^	10.23 ± 0.15 ^e^

Values are presented as mean ± standard deviation (*n* = 3). Different lowercase letters (a–e) in the same column indicate significant differences (*p* < 0.05).

## Data Availability

The original contributions presented in the study are included in the article, further inquiries can be directed to the corresponding author.

## Abbreviations

**Abbreviation**	**Name**
HEC	Hydroxyethyl cellulose
SRBP	Sulfated rice bran polysaccharides
UV	Ultraviolet
RBP	Rice bran polysaccharides
WVP	Water vapor permeability
*L**	Luminosity
*a**	Redness/greenness
*b**	Yellowness/blueness
Δ*E*	Total color difference
TS	Tensile strength
EB	Elongation at break
SEM	Scanning electron microscopy
FT-IR	Fourier transform infrared spectrometry
DSC	Differential scanning calorimetry
TGA	Thermogravimetric Analysis
DPPH	2,2-Diphenyl-1-picrylhydrazyl
·O_2_^−^	Superoxide anion radical
*E. coli*	*Escherichia coli*
*S. aureus*	*Staphylococcus aureus*
To	Onset temperature
Td	Peak temperature
ΔH	Enthalpy change
